# A Randomized Controlled Trial Comparing Intravenous Lidocaine Infusion With Thoracic Epidural for Perioperative Analgesia and Quality of Recovery After Surgery in Laparoscopic Left-Sided Colon and Sphincter-Sparing Rectal Resection Surgery

**DOI:** 10.7759/cureus.23758

**Published:** 2022-04-02

**Authors:** Namitha Birur Jayaprabhu, Jyothi Avula, Tony T Chandy, Gigi Varghese, Bijesh Yadav, Grace Rebekah

**Affiliations:** 1 Department of Anaesthesiology, Christian Medical College Vellore, vellore, IND; 2 Anaesthetics, New Cross Hospital, Royal Wolverhampton NHS Trust, Wolverhampton, GBR; 3 Department of Anaesthesiology, Christian Medical College Vellore, Vellore, IND; 4 Department of Colorectal Surgery, Royal Stoke University Hospital (RSUH) University Hospitals of North Midlands NHS Trust (UHNM), Stoke On Trent, GBR; 5 Department of Colorectal Surgery, Christian Medical College Vellore, Vellore, IND; 6 Department of Biostatistics, Christian Medical College Vellore, Vellore, IND

**Keywords:** enhanced recovery, quality of recovery, perioperative analgesia, length of hospital stay, laparoscopy, epidural, intravenous lidocaine infusion, colon and rectal surgery

## Abstract

Background

Protocols for Enhanced Recovery after Surgery (ERAS) have been constantly evolving, and the best method of managing perioperative pain, especially in laparoscopic surgeries, is still debatable. The primary goal of these protocols is to steer toward opioid-sparing analgesia. Intravenous lidocaine, which has both analgesic and anti-inflammatory properties, may improve the overall recovery of patients.

Objectives

The aim of this randomized controlled trial was to compare the efficacy of intravenous lidocaine infusion (IVL) with thoracic epidural analgesia (TEA) in the management of perioperative pain and recovery in the laparoscopic left-sided colon and sphincter-sparing rectal surgery.

Methods

In this study, 37 patients were randomized to either the IVL group or the TEA group. IVL infusion was started before the surgical incision and stopped 30 minutes after transferring the patient to the postanesthesia care unit (PACU). Postoperative pain scores, opioid consumption, rescue analgesic doses, quality of recovery scores, time to discharge, and adverse events were recorded prospectively. Data were analyzed using two independent sample t-test and paired t-test, with p < 0.05 taken as statistically significant.

Results

The mean difference of overall NRS (numerical rating scale) pain scores in the ward was significantly higher in the IVL group as compared to the TEA group, which was 3.58 (2.29) vs 2.23 1.95) (p < 0.001). The IVL group required more mean rescue opioid boluses than the TEA group, which was 11.36 (8.684) vs 5.96 (6.215) (p < 0.001). However, both IVL and TEA groups had similar pain scores intraoperatively and in the PACU.

Conclusions

TEA provides better analgesia and decreased opioid requirements compared to intravenous lidocaine during the 24-hour period in the ward after laparoscopic left-sided colon and sphincter-sparing rectal surgery, although there was no difference in the quality of recovery between IVL and TEA groups.

## Introduction

Colorectal cancer is the third most common cancer in the world after lung and breast cancer. Close to 1.8 million new cases of colorectal cancer were diagnosed in 2018 [1]. Depending on the stage of cancer, surgical resection remains the main treatment modality. A successful surgical outcome is the result of an amalgamation of surgical, patient, and anesthetic aspects, of which adequate pain control, mitigating stress response, and improving the speed of recovery are of utmost importance [2]. The use of laparoscopy for such resections has increased and is now globally accepted due to substantial short-term and long-term benefits such as decreased length of hospital stay, less morbidity, decreased postoperative pain, and improved quality of recovery when compared to open surgical approaches [3]. Fast-track surgery and acute rehabilitation with effective postoperative analgesia help accelerate postoperative recuperation and limit the duration of hospital stay [4,5]. It has been shown that after laparoscopic surgery, pain experienced is of the same severity as in open surgery during the first 24 hours and of maximal intensity during the first 4 hours following surgery, which is frequently undertreated [6]. The optimal perioperative analgesic protocol for laparoscopic colorectal operations is still contentious [7].

Even though studies have demonstrated the superiority of thoracic epidural in open abdominal operations, evidence regarding its benefit in laparoscopic operations is unconvincing [7,8]. A review conducted by Joshi et al. indicated comparable pain scores in the thoracic epidural and nonepidural groups in laparoscopic colorectal surgery. Due to a higher risk:benefit ratio, the utility or routine epidural analgesia should be evaluated in each individual case [9]. The effects of intravenous lidocaine in comparison to thoracic epidural for analgesia postoperatively at different time points and overall opioid requirements along with various adverse effects are still unclear [10]. Most of the studies that have shown that intravenous lidocaine decreases postoperative opioid consumption are conducted in heterogeneous populations, and hence there is a need for looking specifically at advanced laparoscopic operations with similar surgical incisions [11]. There is evidence suggesting differing analgesic efficacy in the context of different surgical procedures [12]. Our study population included only laparoscopic left-sided colon resections and sphincter-sparing rectal resection due to similarity in port placements and specimen extraction incision. We used the QoR-15 score (quality of recovery score), which analyzes the global recovery of patients by assessing five dimensions of health, which include, emotional state, physical comfort, physical independence, pain, and psychological support [13].

The primary objective of this randomized controlled trial (RCT) was to assess postoperative analgesia using NRS (numerical rating scale) scores, which were recorded at 5 minutes, 30 minutes, 1 hour, 1.5 hours, and 2 hours in the postanesthesia care unit (PACU) and again in the surgical ward at arrival and at 2, 4, 8, 12, and 24 hours between intravenous lidocaine infusion (IVL) and thoracic epidural analgesia (TEA) in advanced laparoscopic colorectal resections. An assessment of postoperative pain by itself does not reflect the overall quality of recovery of a patient. Hence, we used the QoR-15 questionnaire to quantify postoperative recovery [14].

## Materials and methods

This study was carried out over a period of 14 months from June 2017 to August 2018 in the Department of Anaesthesiology at the Christian Medical College Vellore in South India. Before the study was initiated, approval was obtained from the Institutional Review Board (IRB Min. reference number: 10542 dated 20.03.2017). This study was registered with CTRI (Clinical Trial Registry of India) with the registration number CTRI/2018/02/011751.

**Figure 1:**
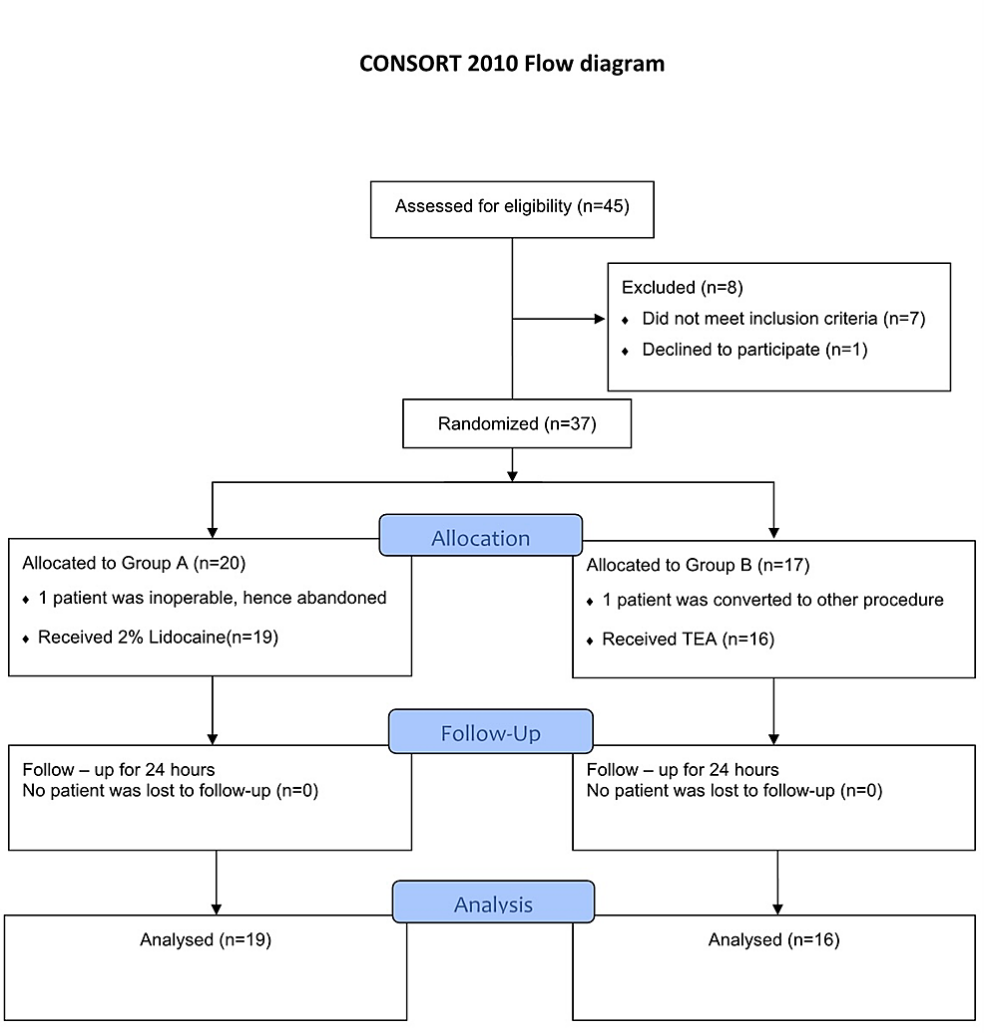
CONSORT flow diagram CONSORT, Consolidated Standards of Reporting Trials Statistical analysis

Participants were recruited for the study after obtaining written informed consent by the principal investigator. It was a prospective, parallel-group, randomized, non-blinded, clinical trial designed in accordance with the CONSORT (Consolidated Standards of Reporting Trials) 2010 guidelines (Figure 1).

A total of 37 patients with American Society of Anaesthesiologists physical status score (ASA) I or II, aged more than 18 years, and undergoing elective laparoscopic left-sided colon and sphincter-sparing rectal resections were recruited for the study. Two patients were subsequently excluded (one patient had to undergo an open operation and the other patient was inoperable as this study included laparoscopic bowel resection patients only). Patients with ASA status III and IV, history of allergy to a local anesthetic, seizure disorder, psychiatric disorder, treatment with antiarrhythmic drugs, and steroids and chronic opioid use were excluded. We used the QoR-15 score, which analyzes the global recovery of patients by assessing five dimensions of health including emotional state, physical comfort, physical independence, pain, and psychological support. A baseline QoR-15 score was documented preoperatively in the ward for all the recruited patients, and they were familiarized with the NRS pain scores and the use of patient-controlled analgesia (PCA) pump (CADD-Legacy® PCA Ambulatory Infusion Pump, Model 6300, Smiths Medical, Kent, UK).

Patients were randomly allocated to group A (IVL group) and group B (TEA group) using computer-generated permuted block randomization, and sequentially numbered, sealed, opaque envelopes were opened in the operation theater by the respective anesthetist who was not involved directly in the study. Patients were then allocated to the respective groups based on the randomization.

Patients in the IVL group (group A, n=19) received a bolus of 2 mg kg^-1^ intravenous lidocaine just before induction of anesthesia. This was followed by a continuous infusion of 2 mg/kg/hour of intravenous lidocaine, which was stopped 30 minutes after the patient was shifted to the PACU. Patients in the epidural group (group B, n = 16) had a thoracic epidural catheter placed at T8-10 intervertebral spaces before induction of general anesthesia and a bolus of 3-5 mL of 0.25% bupivacaine was given, followed by an infusion of the same at 5-8 mL/hour during the operation. In the PACU, the epidural was continued as an infusion of 0.1% bupivacaine with 2 ug of fentanyl at 4-5 mL/hour. This was stopped after 48 hours postoperatively.

During the operation, standard ASA monitoring was applied, and all patients were induced with intravenous fentanyl 2 ug/kg, propofol 2 mg/kg, and vecuronium 0.1 mg/kg. After securing the airway with an endotracheal tube, anesthesia was maintained with isoflurane adjusted to a MAC (minimum alveolar concentration) of 0.8-1.0 and mechanically ventilated with air-oxygen mixture (50% FiO_2_). Intravenous paracetamol 15-20 mg/kg was given after induction. Supplemental doses of intravenous fentanyl (0.5-1 ug/kg) were given to attenuate the pain response (when there was a 20% increase in intraoperative blood pressure and heart rate from the baseline). Intraoperative hemodynamics was maintained with intravenous fluids at a rate of 5-8 mL/kg/hour. Intravenous morphine and tramadol were given if there was a further pain response as per the discretion of the anesthesiologist involved with the surgery in the operation theater. Patients were extubated after meeting the extubation criteria and were shifted to PACU. NRS scores were recorded at 5 minutes, 30 minutes, 1 hour, 1.5 hours, and 2 hours in the PACU.

All patients received PCA with intravenous fentanyl 20 ug per bolus and a lockout period of 15 minutes through the CADD PCA pump for 24 hours postoperatively. Intravenous paracetamol 20 mg/kg every 6 hours and intravenous diclofenac 50mg twice daily were given to all patients till the second postoperative day. In the ward, all patients were followed up and the following parameters were recorded: NRS at arrival and at 2, 4, 8, 12, and 24 hours, Total opioid consumption (total number of fentanyl boluses from the CADD PCA pump) at arrival and at 2, 4, 8, 12, and 24 hours, QoR 15 score after 24 hours postoperatively, duration of hospital stay, and incidence of any perioperative adverse events in both IVL and TEA groups.

Statistical analysis

The sample size was calculated based on a retrospective study by Tikuišis et al. [15]. A maximum standard deviation (SD) was assumed to be 1.6 in both the groups with a clinically expected difference in NRS scores to be 1.5 units with alpha error fixed at 5% with power at 80%. This analysis indicated that we needed to study at least 18 patients in each arm who underwent laparoscopic left-sided colon and sphincter-sparing rectal surgeries.

Data were entered into the Epidata software, and analysis was conducted using SPSS Version 21.0 (IBM Corp., Armonk, NY). Descriptive statistics were reported using mean (SD) for continuous variables. Categorical data were reported using frequency and percentage. Bar graphs and line diagrams were used for the graphical representation of data. Based on the normality of the data, either parametric t-test or nonparametric Mann-Whitney test was applied. Comparison of means between the two arms for continuous variables was done using two independent sample t-test. Association between the arms with respect to the categorical variable was reported using chi-square or Fisher's exact test. The primary outcome was to assess postoperative pain. A two-sample T-test was applied to calculate the significance between the two arms. The two-sample T-test was also used to analyze demographics (age, weight, height, BMI), baseline vitals, rescue analgesic dose given, and change in the QoR-15 scores. All tests were two-sided at α=0.05 level of significance.

## Results

Demographic data, physical characteristics, and type and duration of surgery were comparable in both groups of patients. There was no difference in time to extubation calculated from the time of discontinuing inhalational agents in both groups (Table 1). The mean duration of lidocaine infusion was 368.9 ± 75.9 minutes. None of the patients in group A (IVL group) had any adverse events related to the infusion; however, one patient had occasional ventricular ectopics, which was attributed to the insertion of the PICC (peripherally inserted central catheter) line as it resolved completely upon readjusting the line. In group B (TEA group), there were two patients with failed epidurals, one patient with catheter displacement in the ward, and one patient needing to be catheterized for urinary retention (Table 1). There was no difference in the length of hospital stay in both groups (group A: 9.3 ± 7.9; group B: 8.4 ± 3.8 days) (Table 1).

**Table 1:** Patient characteristics in study groups. (A) Intravenous lidocaine. (B) Thoracic epidural analgesia. Data are represented as mean (SD) or as number (percentage) ASA, American Society of Anesthesiologists; BMI, body mass index

Clinical variable	Group A (n=19)	Group B (n=16)
Age (year)	47.7 ± 15.8	51.1 ± 12.4
Gender (male:female	13 (68.4%):6 (31.6%)	11 (68.8%):5 (31.2%)
ASA I	11 (57.9%)	7 (43.8%)
ASA II	8 (42.1%)	9 (56.2%)
Weight (kg)	67.5 ± 12.0	60.7 ± 5.4
Height (cm)	162.5 ± 10.9	163.2 ± 7.8
BMI	25.5 ± 4.0	23.0 ± 2.7
Hypertension	6 (31.6%)	5 (31.2%)
Diabetic	2 (10.5% )	2 (12.5%)
Duration surgery (minutes)	281.0 ± 73.9	234.4 ± 74.6
Time to tracheal extubation (minutes)	14.5 ± 5.8	14.0 ± 3.9
Duration of hospital stay (days)	9.3 ± 7.9	8.4 ± 3.8
Adverse effects	0	4

**Figure 2:**
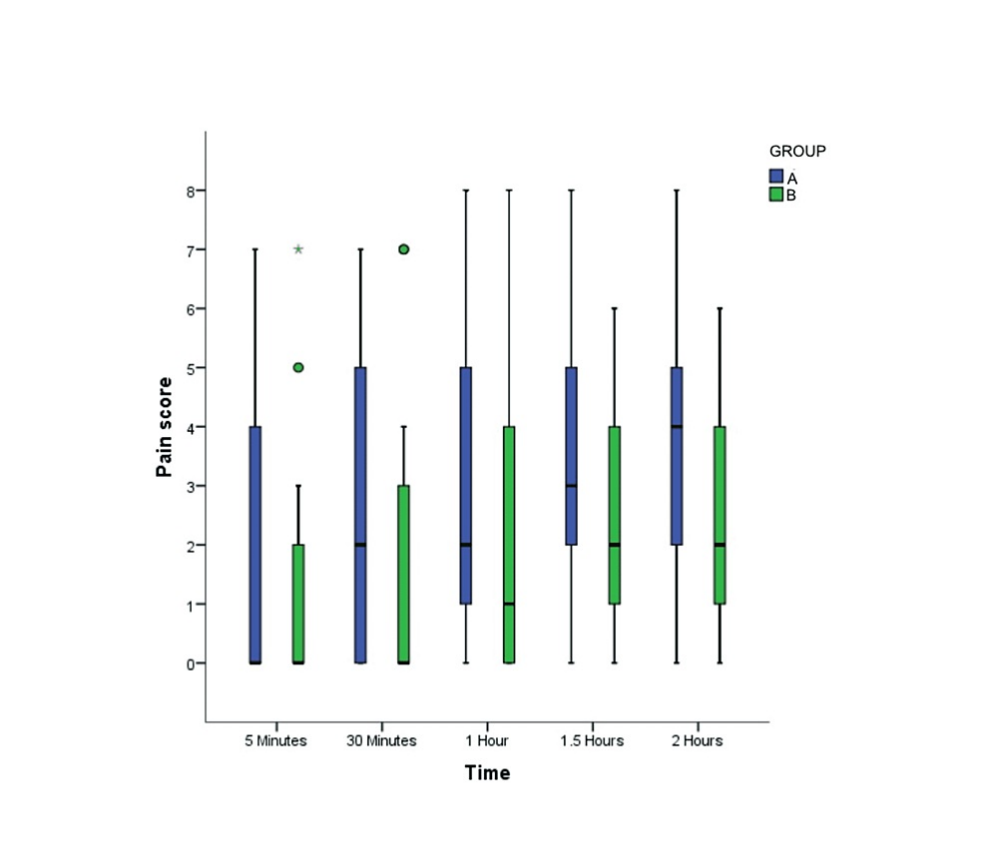
Postoperative NRS pain scores in the PACU at different time intervals in the study groups (A) intravenous lidocaine and (B) thoracic epidural analgesia. Data are represented as mean ± SD. There was no significant difference in the pain scores between both the study groups. NRS, numeric rating scale; PACU; postanesthesia care unit

**Figure 3:**
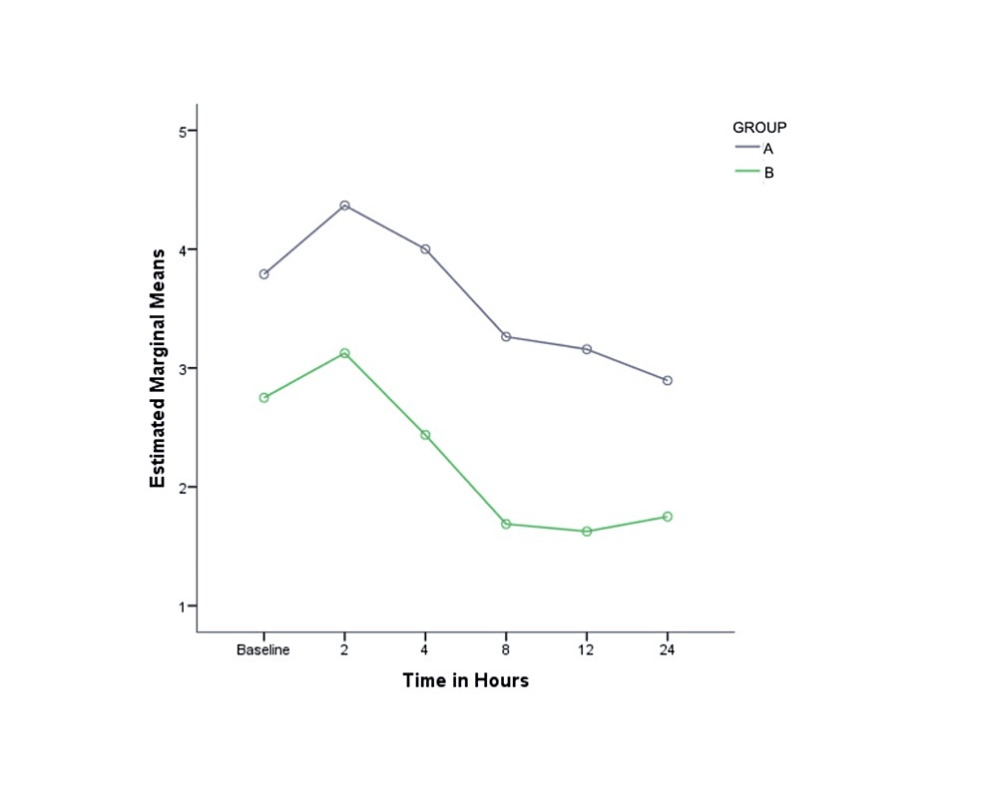
NRS pain scores at arrival and at 2, 4, 8, 12, and 24 hours to the postoperative ward respectively in the study groups (A) intravenous lidocaine and (B) thoracic epidural analgesia. Values are represented as mean (SD). NRS pain scores were significantly higher at 4, 8,12, and 24 hours in group A (p < 0.05) as compared to group B. NRS, numeric rating scale

NRS pain scores documented between the study groups in the PACU up to 2 hours postextubation did not show any statistically significant difference (Figure 2), and most of the patients (74.6% in group A and 87.5% in group B ) had NRS pain scores that were less than 4. However, the pain scores were significantly higher in group A (IVL) from 4 to 24 hours after shifting to the ward (p = 0.047 at 4 hours, 0.018 at 8 hours, 0.015 at 12 hours, and 0.043 at 24 hours; Figure 3).

The average dose of fentanyl (in micrograms) that patients received through the CADD PCA pump was significantly greater at all time intervals after arrival in the ward till 24 hours in group a than group B (p < 0.05; Table 2). There was no difference in the cumulative amount of analgesics used during the intraoperative period between the two groups (Table 3).

**Table 2:** Mean PCA fentanyl consumption (mcg) in the postoperative ward at different time intervals in the study groups (A) intravenous lidocaine and (B) thoracic epidural analgesia. Values are presented as mean ± SD. There was a statistically significant increase in fentanyl consumption in group A as compared to group B. *p < 0.05 PCA, patient-controlled analgesia

	Group A (N=19) (mean of PCA fentanyl in mcg)	Group B (N=15) (mean of PCA fentanyl in mcg)	p-value
Arrival to 2 hours	67.4 ± 35.4	29.3 ± 45.9	0.010*
2–4 hours	75.8 ± 43	28 ± 39.9	0.002*
4–8 hours	63.2 ± 52.2	38.7 ± 47.5	0.167
8–12 hours	79 ± 56	30.7 ± 45.3	0.011*
12–24 hours	79 ± 61	38.7 ± 33.4	0.028*

**Table 3:** Cumulative intraoperative analgesic consumption in the study groups (A) intravenous lidocaine and (B) thoracic epidural analgesia. Values are expressed as mean ± SD. NSAIDs, non-steroidal anti-inflammatory drugs

	Group A (N = 19)	Group B (N = 16)	p-Value
Fentanyl (mcg)	207.9 ± 55.0	183.8 ± 55.0	0.205
Morphine (mg)	4.4 ± 2.7	4.8 ± 1.2	0.766
NSAIDs (mg)	68.7 ± 12.5	50.0 ± 0.0	0.272
Tramadol (mg)	75 ± 28.9	100	0.495

**Figure 4:**
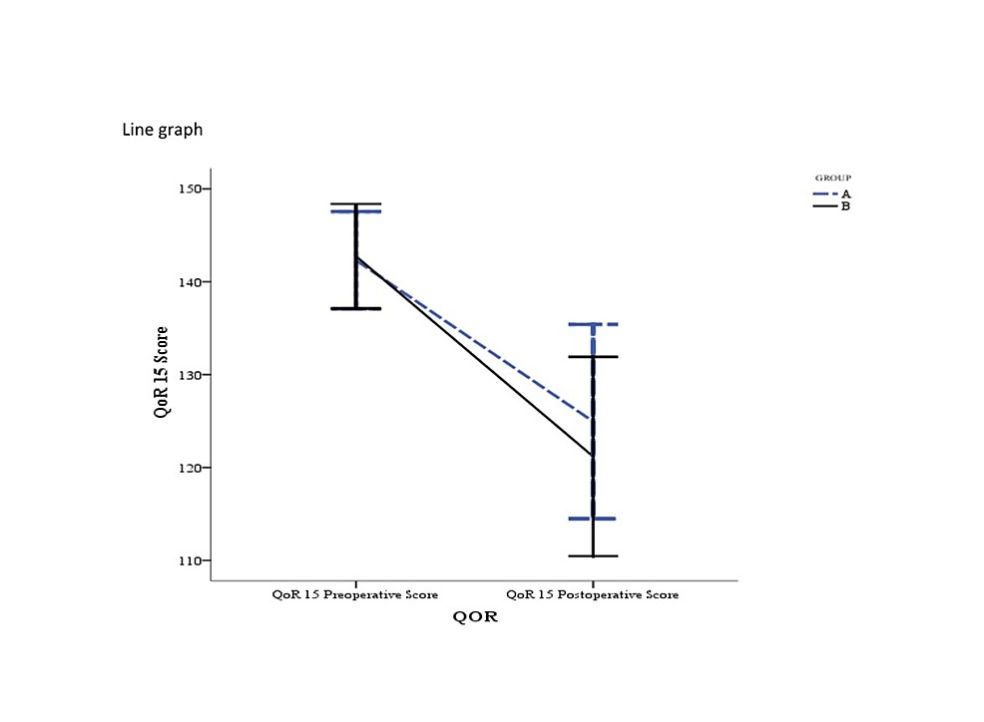
The total QoR-15 scores per time point. The data are presented as mean with SD. The percentage and absolute decrease between preoperative and postoperative QoR-15 scores were not statistically significant between the two groups: (A) intravenous lidocaine and (B) thoracic epidural analgesia. QoR, Quality of Recovery

The percentage decrease in the QoR-15 scores in the postoperative period assessed 24 hours after surgery was less in group A as compared to group B, though the difference was not statistically significant (11.6% vs. 14.4%, p = 0.508, Figure 4).

## Discussion

Overall, TEA was statistically superior to IVL for postoperative analgesia in this study. Perioperative IVL for a short duration produced analgesia comparable to TEA in the intraoperative period (based on analgesic consumption in Table 3) and up to 2 hours in the PACU, but it failed to translate the same benefits into the remaining postoperative period in the ward. Whether superior analgesic effect of TEA beyond immediate postoperative period is due to not continuing infusional lignocaine is debatable as quality of recovery and postoperative days were comparable. Despite the significantly higher mean PCA fentanyl consumption (mcg) and higher postoperative pain scores in the ward in the IVL group, there was no difference in the quality of recovery assessed after 24 hours postsurgery on postoperative day 1 and in the duration of hospital stay in both groups of patients.

There is robust evidence of high-quality pain relief and a protective role against pulmonary complications with epidural analgesia [16]. However, epidural analgesia does not seem to offer any additional clinical benefits in patients undergoing laparoscopic colorectal surgery [17]. It also has to be noted that although advanced laparoscopic operations are associated with minimal abdominal incisions, the pain is at maximum intensity within the first 4 hours after surgery and hence we need to focus on the role of other modalities on optimal pain relief in the immediate postoperative period. Cost-effectiveness along with prolonged hospital stay and higher incidence of urinary infections that are associated with epidural insertion are major concerns [18]. Failure rates of epidural insertion are persistently high at around 22-32%, which is one of the limiting factors for its routine use [19].

Various studies have explored the antinociceptive, antihyperalgesic [20], and anti-inflammatory action of lidocaine [21] attributable to its action on intracellular G protein Y coupled receptors, complements, and cytokines [22,23], though the exact mechanism remains largely unknown [24]. However, there is still a lack of consensus among clinicians with regard to a befitting dose regimen that can be safely used in patients undergoing advanced laparoscopic operations. Khan et al. conducted a meta-analysis to determine an appropriate duration of IVL in both open and laparoscopic bowel surgeries [25] and concluded that continuing the infusion beyond 60 minutes offered no added benefit. Based on these data, we continued lidocaine infusion till 30 minutes after shifting the patient to PACU. An infusion dose of 2 mg kg^-1^ hour^-1^ was used in our study based on the safe dosage limits of lignocaine infusion as demonstrated in prior studies [26,27].

Terkawi et al. concluded that the pain scores in the lidocaine group were comparable to the epidural group but inferior with respect to opioid consumption in major abdominal surgeries. They also suggested that intraoperative lidocaine infusion appears to play a compelling role in the postoperative effect irrespective of the duration of postoperative infusion [28,29]. This is not substantiated in our study where the analgesic effect was limited to an acute postoperative interval, which was also noted in the study by Staikou et al. [30]. There was a significant increase in pain scores and opioid consumption in our study after the patient was shifted from PACU to the ward, demonstrating gradual fade-in analgesia in the IVL group. Whether prolonging IVL up to and beyond 24 hours helps in better analgesia and decreased opioid consumption in the ward is yet to be substantiated, although some studies have shown varying results [31,32]. The comparable hemodynamics and analgesic effect in both groups during the intraoperative period demonstrates that perioperative use of intravenous lidocaine is an effective method of analgesia during laparoscopic surgeries, which could potentially be used as an alternative to TEA.

Multimodal benefits and safety of intravenous lidocaine perioperatively have been elucidated in various studies in bowel surgeries [10,11,33]. It is interesting to note that the pain scores in both the study groups were predominantly less than or equal to 4 on the NRS scale and there was no difference in the quality of recovery and duration of hospital stay between the two groups, suggesting its multiple benefits.

To our knowledge, this is one of the first few randomized controlled studies comparing IVL with TEA, specifically in this group of patients, which assessed analgesia at different time intervals along with the overall quality of recovery of the patient.

Limitations

Our study was a single-center RCT that would confer a certain level of bias to the study. Surgical and anesthetic experience was not factored into the study due to the heterogeneous surgical and anesthetic teams involved. Prior abdominal surgeries and other technical difficulties should have been captured and factored into the study for a better comparison of the results. Enhanced Recovery after Surgery (ERAS) protocol was not strictly followed during the study, which could have affected the absolute values of outcome parameters. Continuing infusional lignocaine for the same duration as TEA may have affected the study results in the late postoperative period.

## Conclusions

In conclusion, TEA may be superior to IVL for postoperative analgesia in patients undergoing laparoscopic left-sided colon and rectal resections. However, analgesia with intravenous lidocaine is comparable to TEA intraoperatively and in the PACU. Lidocaine can be used as an adjunct in multimodal analgesia in laparoscopic surgeries where epidural analgesia is not warranted. Given that laparoscopic colorectal surgeries are becoming widely used as a standard of care, we recommend future multicentric randomized controlled studies with intravenous lidocaine to formulate optimal duration of infusion.
